# A HIV-1 Tat mutant protein disrupts HIV-1 Rev function by targeting the DEAD-box RNA helicase DDX1

**DOI:** 10.1186/s12977-014-0121-9

**Published:** 2014-12-14

**Authors:** Min-Hsuan Lin, Haran Sivakumaran, Alun Jones, Dongsheng Li, Callista Harper, Ting Wei, Hongping Jin, Lina Rustanti, Frederic A Meunier, Kirsten Spann, David Harrich

**Affiliations:** QIMR Berghofer Medical Research Institute, Herston, Queensland Australia; Australian Infectious Disease Research Centre, The University of Queensland, St. Lucia, Queensland Australia; Institute for Molecular Bioscience, The University of Queensland, St. Lucia, Queensland Australia; Queensland Brain Institute, The University of Queensland, St. Lucia, Queensland Australia; School of Chemistry and Molecular Biosciences, The University of Queensland, St. Lucia, Queensland Australia

**Keywords:** HIV-1, Rev, Tat, Nullbasic, Helicase, DDX1, RNA export

## Abstract

**Background:**

Previously we described a transdominant negative mutant of the HIV-1 Tat protein, termed Nullbasic, that downregulated the steady state levels of unspliced and singly spliced viral mRNA, an activity caused by inhibition of HIV-1 Rev activity. Nullbasic also altered the subcellular localizations of Rev and other cellular proteins, including CRM1, B23 and C23 in a Rev-dependent manner, suggesting that Nullbasic may disrupt Rev function and trafficking by intervening with an unidentified component of the Rev nucleocytoplasmic transport complex.

**Results:**

To seek a possible mechanism that could explain how Nullbasic inhibits Rev activity, we used a proteomics approach to identify host cellular proteins that interact with Nullbasic. Forty-six Nullbasic-binding proteins were identified by mass spectrometry including the DEAD-box RNA helicase, DDX1. To determine the effect of DDX1 on Nullbasic-mediated Rev activity, we performed cell-based immunoprecipitation assays, Rev reporter assays and bio-layer interferometry (BLI) assays. Interaction between DDX1 and Nullbasic was observed by co-immunoprecipitation of Nullbasic with endogenous DDX1 from cell lysates. BLI assays showed a direct interaction between Nullbasic and DDX1. Nullbasic affected DDX1 subcellular distribution in a Rev-independent manner. Interestingly overexpression of DDX1 in cells not only restored Rev-dependent mRNA export and gene expression in a Rev reporter assay but also partly reversed Nullbasic-induced Rev subcellular mislocalization. Moreover, HIV-1 wild type Tat co-immunoprecipitated with DDX1 and overexpression of Tat could rescue the unspliced viral mRNA levels inhibited by Nullbasic in HIV-1 expressing cells.

**Conclusions:**

Nullbasic was used to further define the complex mechanisms involved in the Rev-dependent nuclear export of the 9 kb and 4 kb viral RNAs. All together, these data indicate that DDX1 can be sequestered by Nullbasic leading to destabilization of the Rev nucleocytoplasmic transport complex and decreased levels of Rev-dependent viral transcripts. The outcomes support a role for DDX1 in maintenance of a Rev nuclear complex that transports viral RRE-containing mRNA to the cytoplasm. To our knowledge Nullbasic is the first anti-HIV protein that specifically targets the cellular protein DDX1 to block Rev’s activity. Furthermore, our research raises the possibility that wild type Tat may play a previously unrecognized but very important role in Rev function.

**Electronic supplementary material:**

The online version of this article (doi:10.1186/s12977-014-0121-9) contains supplementary material, which is available to authorized users.

## Background

The human immunodeficiency virus type-1 (HIV-1) Rev *trans-activator* is required for virus replication. It is expressed from the multiply spliced (~2 Kb) viral transcript and mediates the expression of viral structural, enzymatic and accessory proteins from the unspliced (~9 Kb) and singly spliced (~4 Kb) viral transcripts [1]. Rev is a 116 amino acid protein and can be divided into three discrete function domains. The Rev RNA-binding domain (RBD, amino acids 35–51) is an arginine-rich motif that serves as the nucleolar localization signal (NLS), which can be recognized by the cellular importin β-like import receptors and nucleophosmin (NPM), also known as B23 [2–5]. This region also specifically interacts with a stem loop RNA sequence called Rev response element (RRE), which is located within the *env* gene among unspliced and singly spliced HIV-1 mRNAs [6,7]. The activation domain (amino acid 77–83) is a leucine-rich motif that acts as a nuclear export signal (NES) that directly interacts with cellular chromosome region maintenance 1(CRM1), also known as exportin 1 (XPO1), in the presence of RanGTP [8–11]. Regions flanking the RBD constitute the multimerization domain (amino acids 12–33 and 51–60). It has been demonstrated that formation of the HIV-1 Rev:RRE protein complex [also called Rev ribonucleoprotein (RNP) complex] requires the recruitment of multiple Rev monomers [12–14]. Since Rev contains both NLS and NES, it acts as a shuttling protein that constantly traffics between the nucleus and the cytoplasm. In HIV-1 infected cells, Rev binds to unspliced and singly spliced HIV-1 mRNAs via their RRE to form a Rev RNP complex with CRM1 and other cellular components in the nucleolus, then CRM1 directs the whole complex through the nuclear pore to the cytoplasm [10,15,16]. The Rev RNP complex is disassembled in the cytoplasm, allowing translation to begin. Cytoplasmic Rev is then recognized by the importin β-like import receptors, such as importin β and transportin 1, and transported back to the nucleus [3,5,17]. Once Rev enters the nucleus, B23 binds to Rev’s NLS in the RBD and facilitates import of Rev to the nucleolus for reformation of the Rev RNP complex [4]. In addition to Rev’s major function in promoting nuclear export of incompletely spliced viral transcripts, other activities in integration, translation and encapsidation have been described [18–20].

DEAD (Asp - Glu - Ala - Asp)-box helicases form the largest family of RNA helicases and are conserved in bacteria, archaea and eukaryotes [21]. They are associated with many levels of RNA function including transcription, pre-mRNA splicing, ribosome biogenesis, RNA trafficking, RNA decay and translation initiation [22,23]. Although HIV-1 does not encode for an RNA helicase, a number of cellular DEAD-box RNA helicases, including DDX1, DDX3, DDX5/p68, DDX17, DDX21, DDX24, DDX36, DDX47 and DDX56, have been identified to play crucial roles in the HIV-1 replication cycle, particularly in the regulation of Rev function [24]. DDX1 directly interacts with the multimerization domain of HIV-1 Rev protein to promote Rev oligomerization on the RRE [25]. Overexpression of DDX1 in HIV-1 infected cells results in increased virus production, while downregulation of DDX1 by RNAi not only inhibits viral replication but also unexpectedly alters the subcellular localization of Rev from predominantly nucleolus to nucleus and cytoplasm, resulting in inhibition of nuclear export of incompletely spliced viral mRNAs [26,27]. Of note, low DDX1 levels in primary human astrocytes have effects on Rev subcellular localization similar to DDX1 RNAi [28]. Similar to DDX1, DDX3, known as a nucleocytoplasmic shuttle protein, directly binds to CRM1 and mediates Rev-dependent viral mRNA transport [29]. Knockdown of endogenous DDX3 using short hairpin RNA (shRNA) or expression of DDX3 transdominant-negative mutant protein suppresses nuclear export of incompletely spliced HIV-1 mRNAs and viral replication [29,30]. In addition to modulation of Rev function, DDX3 was reported to directly interact with the HIV-1 Tat protein to facilitate Tat function and HIV-1 mRNA translation [31,32]. Recent studies further indicate that DDX5, DDX17, DDX21, DDX24, DDX36, DDX47 and DDX56 all associate with the HIV-1 Rev protein and cooperate to regulate Rev function [33,34].

Our previous studies described a two-exon HIV-1 Tat mutant termed Nullbasic, created by replacing the entire basic domain of wild-type Tat with glycine and alanine residues, which provides strong protection from HIV-1 infection by potently inhibiting multiple steps of the HIV-1 replication cycle [35]. Nullbasic has at least three anti-HIV-1 activities. It inhibits Tat-mediated transactivation activity, suppresses HIV-1 reverse transcription and reduces steady state levels of unspliced and singly-spliced viral mRNA by the inhibition of Rev activity [35,36]. With respect to Rev, Nullbasic also alters its subcellular localization as well as the subcellular localizations of cellular proteins including CRM1, B23 and C23 in a Rev-dependent manner, suggesting that Nullbasic interferes with a component of the Rev nucleocytoplasmic transport complex required for Rev trafficking and function [35,37]. Here we employed a proteomics approach to identify cellular proteins that interact with Nullbasic. Co-immunoprecipitation assays identified 46 host protein candidates across independent mass spectrometry analyses, including three DEAD-box RNA helicases, DDX1, DDX3 and DDX17. We found that DDX1 interacts with Nullbasic directly leading to the disruption of Rev subcellular localization and resulting in decreased levels of incompletely spliced viral transcripts. Thus, our study provides further evidence of an important role for DDX1 in the maintenance of Rev subcellular localization and function. Interestingly, co-immunoprecipitation showed that wild type Tat also associated with DDX1 and overexpression of Tat in cells could restore Rev mediated export of viral mRNA that had been inhibited by Nullbasic, implying a novel and previously unrecognized function for wild type Tat to support Rev function.

## Results

### Identification of Nullbasic interacting proteins by LC MS/MS

**Figure 1:**
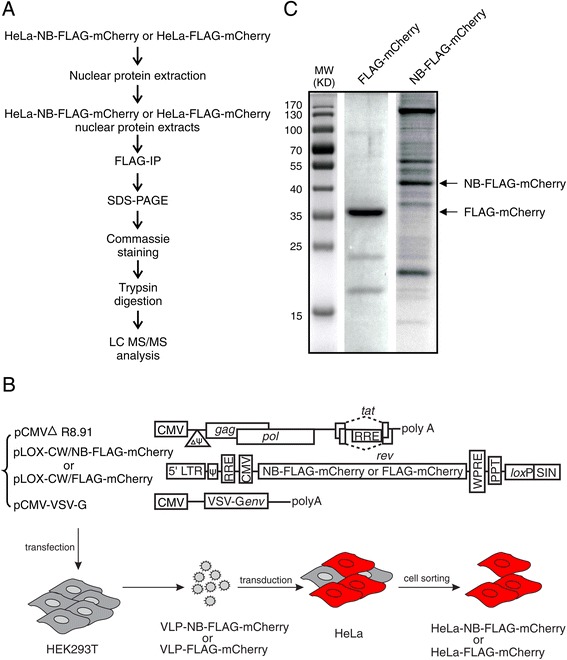
**Proteomic strategy for identifying Nullbasic-interacting proteins from HeLa-Nullbasic-mCherry cells. A**. Overview of the proteomic approach for isolating Nullbasic (NB) binding proteins from cell nuclear extracts. **B**. Schematic diagrams of the VLP expression plasmids and an overview of the methods used to generate lentiviral VLPs. pCMV∆R8.91, pCMV-VSV-G and pLOX/CW-Nullbasic-FLAG-mCherry or pLOX/CW-FLAG-mCherry were co-transfected into HEK293T cells to produce VLPs containing either Nullbasic-FLAG-mCherry lentivector genome (VLP-NB-mCherry) or the control FLAG-mCherry genome (VLP-FLAG-mCherry). VLP-NB-mCherry and VLP-FLAG-mCherry were then transduced into HeLa cells in order to create cell lines stably expressing either the NB-mCherry proteins or FLAG-mCherry proteins. **C**. Nuclear protein lysates expressing NB-mCherry or FLAG-mCherry were separated by SDS-PAGE and protein bands were stained with Coomassie dye.

In this study, we focused on nuclear Nullbasic interactors as our previous studies indicated that Nullbasic disrupted the nucleolar colocalization of HIV-1 Rev with CRM1, B23 and C23 but did not affect importin β- and transportin 1-mediated Rev nuclear import pathways [37], suggesting that Nullbasic interferes with specific components of a Rev nuclear complex. The experimental strategy used to identify protein complexes associated with Nullbasic is illustrated in Figure 1A. HeLa cells were transduced with lentiviral vectors that could express either Nullbasic-FLAG-mCherry or FLAG-mCherry protein. For each transduction, mCherry positive cells were collected by FACS and HeLa stable cell lines expressing each protein were established (Figure 1B).

Nuclear fractions were extracted and the purity was analyzed by western blotting [see Additional file 1]. Nuclear Nullbasic-FLAG-mCherry and FLAG-mCherry interacting protein complexes were immunoprecipitated using anti-FLAG beads. Following extensive washes, immunoprecipitated protein complexes were eluted, separated by SDS-PAGE and stained with Coomassie dye (Figure 1C). Protein bands were sliced, digested with trypsin and analysed by LC MS/MS. Protein identification was performed with ProteinPilot 4.0 software (AB SCIEX) against the UniProt database restricted to *Homo sapiens*. Proteins were considered “identified” if more than two peptides identified with a >95% confidence and a < 1% global false discovery rate (FDR). A total of 379 proteins were detected from the NB-FLAG-mCherry nuclear fractions, of which 333 were only detected in a single experiment or detected in the control immunoprecipitations and therefore excluded as “significant”. In the final analysis, 46 proteins were considered “significant” as they were detected at least twice across three independent experiments [see Additional file 2]. These 46 identified proteins could be classified into 6 broad functional groups: RNA splicing/transport factors, folding/transport proteins, transcription regulators, translation factors, cellular organization/cytoskeleton and cell cycle. Of immediate interest were the DEAD-box RNA helicases DDX1, DDX3 and DDX17 since all of these were identified in each experiment, and associate with HIV-1 Rev and regulate its function.

**Figure 2:**
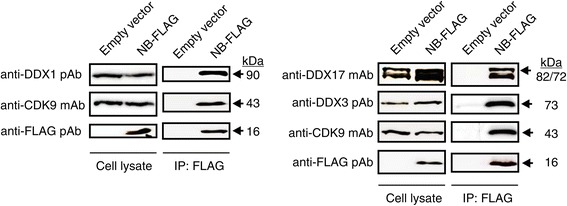
**Interactions of DDX1, DDX3 and DDX17 with Nullbasic** ***in vivo*** **.** HEK293T cells were transfected with either empty vector (pcDNA 3.1) or plasmid expressing NB-FLAG. The FLAG-tagged proteins and their binding partners were immunoprecipated with anti-FLAG beads. Total cell lystes and immunoprecipated proteins were separated by SDS-PAGE and target proteins were detected using anit-FLAG, anti-DDX1, anti-DDX3 and anti-DDX17 antibodies. The anti-CDK9 antibody was used to detect endogenous CDK9 as a positive control for NB interaction. The figure is representative of 3 independent experiments.

To validate the interactions of Nullbasic with DDX1, DDX3 and DDX17, co-immunoprecipitation experiments were performed using HEK293T cells expressing FLAG-tagged Nullbasic (Nullbasic-FLAG) (Figure 2). Immunoprecipitation of Nullbasic-FLAG with anti-FLAG beads did co-immunoprecipitate endogenous DDX1, DDX3 and DDX17 as well as CDK9. The interaction of Nullbasic and CDK9 has been shown previously [37], and served as a positive control. An empty vector (pcDNA 3.1) transfection was included as a negative control. These data confirmed that Nullbasic is able to interact with DDX1, DDX3 and DDX17.

### Nullbasic inhibition of Rev/RRE-dependent mRNA export can be counteracted by overexpression of DDX1

**Figure 3:**
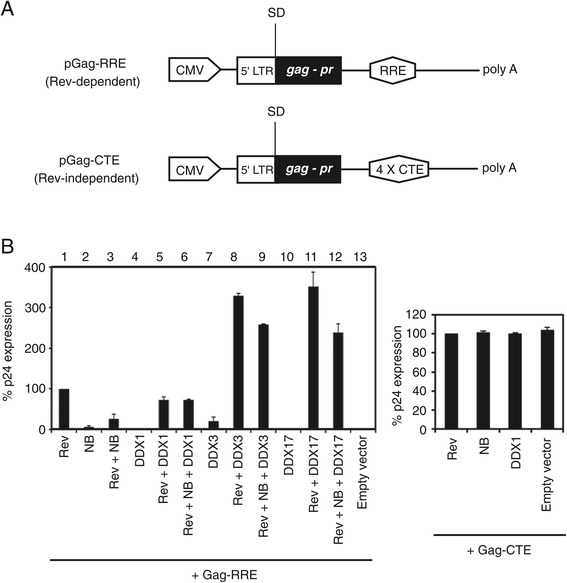
**Overexpression of DDX1 in HeLa cells rescues the Rev/RRE-dependent reporter gene expression suppressed by Nullbasic. A**. Schematic maps of pGag-RRE and pGag-CTE plasmids. **B**. Left: HeLa cells were transfected with appropriate expression plasmids, including pGag-RRE (500 ng), pRSV-Rev (10 ng), Nullbasic (NB, 500 ng), HA-DDX1(1000 ng), HA-DDX3 (1000 ng), DDX17 (1000 ng) and empty plasmid (pcDNA3-HA, 1000 g). Right: HeLa cells were transfected pGag-CTE (500 ng) with pRSV-Rev (10 ng), Nullbasic (NB) (500 ng), HA-DDX1(1000 ng) or empty vector (pcDNA3-HA, 1000 ng). A pcDNA3 plasmid was used to normalize the total amount of transfected plasmids and a luciferase expression plasmid was included to monitor transfection efficiency. After 24 h transfection, the cellular lysates were collected for assay of HIV-1 capsid level and luciferase activity. The p24 production was normalized to the luciferase reporter activity and expressed as a percentage relative to cells transfected with pGag-RRE with pRSV-Rev (left) or pGag-CTE with pRSV-Rev (right). Columns represent the means and standard deviations of transfections performed in triplicate. The experiment was performed 3 times with similar results.

We previously showed that Nullbasic downregulated Rev function and Rev-dependent viral mRNA steady state levels [35]. To test whether this inhibitory effect is due to the interaction of DDX1, DDX3 or DDX17 with Nullbasic, we analyzed translation of Rev-dependent viral mRNA by utilizing pGag-RRE and pGag-CTE reporter plasmids (Figure 3A). Both plasmids are cytomegalovirus (CMV) immediate-early promoter-driven Gag/protease (PR) vectors [38]. The pGag-RRE plasmid contains the RRE that can produce HIV-1 Gag and PR proteins in a Rev-dependent manner, while pGag-CTE plasmid contains 4 copies of the constitutive transport element (CTE) of Mason-Pfizer monkey virus that express Gag and PR proteins in a Rev-independent manner. The Gag-derived proteolytic product capsid (CA) protein was measured in cell lysates by ELISA. As expected, the expression of Rev alone with pGag-RRE produced measurable CA compared to CA expression by pGag-RRE without Rev (Figure 3B, left, lanes 1 and 13). Expression of Nullbasic, HA-tagged DDX1 (HA-DDX1), HA-tagged DDX3 (HA-DDX3), and HA-tagged DDX17 (HA-DDX17) and parental vector (pcDNA3-HA) alone in the Rev-dependent reporter assay observed no or low expression of CA protein (Figure 3B, left, lane 2, 4, 7, 10 and 13). The levels of CA were greatly increased when Rev was coexpressed with HA-DDX3 (Figure 3B, left, lane 8) or HA-DDX17 (Figure 3B, left, lane 11) in the Rev-dependent reporter assay compared to expression of Rev alone (Figure 3B, left, lane 1), which are similar to results seen in previous studies [29,33,39]. Coexpression of Rev with HA-DDX1 (Figure 3B, left, lane 5) reduced CA expression ~ 25% (*p = 0.02*) compared to expression of Rev alone (Figure 3B, left, lane 1). However, coexpression of Rev and Nullbasic in the Rev-dependent reporter assay resulted in a ~ 80% decrease (*p = 0.0004*) of CA expression (Figure 3B, left, lane 3) compared to expression of Rev alone (Figure 3B, left, lane 1). This result confirmed our previous studies that Nullbasic can interfere with Rev function, leading to loss of Rev-mediated viral gene expression [35]. Quite surprisingly, Nullbasic did not alter CA expression when HA-DDX1 was expressed in the cells co-expressing Rev and Nullbasic (Figure 3B, left, comparison of lane 5 and 6). In contrast, the levels of CA were decreased 20% (*p = 0.00005*) and 30% (*p = 0.009*) in the reporter assay coexpressing Nullbasic, Rev and HA-DDX3 (Figure 3B, left, lane 9) or HA-DDX17 (Figure 3B, left, lane 12) compared to coexpression of Rev and HA-DDX3 (Figure 3B, left, lane 8) or HA-DDX17 (Figure 3B, left, lane 11). Expression of Rev, Nullbasic, HA-DDX1and parental vector (pcDNA3-HA) had no effect on CA expression from pGag-CTE (Figure 3B, right). We conclude that overexpression of DDX1 can compensate for the inhibitory effect of Nullbasic on Rev function.

### Nullbasic affects the subcellular distribution of DDX1

**Figure 4:**
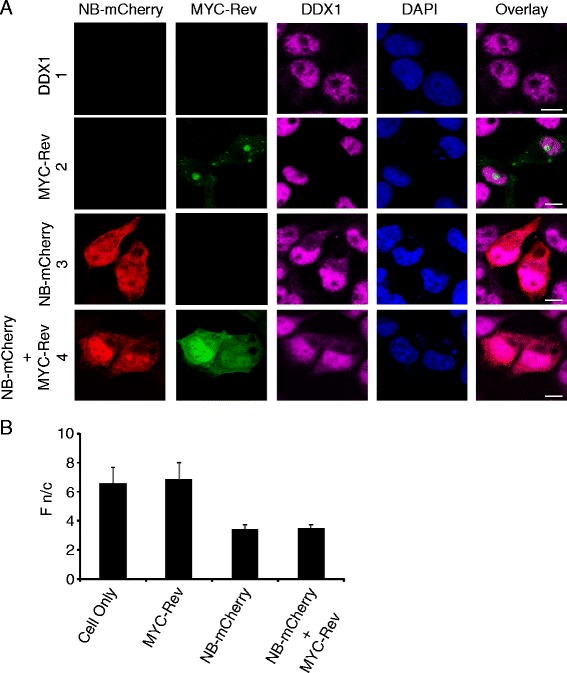
**Nullbasic alters the subcellular localization of DDX1. A**. HeLa cells were transfected with empty vector alone (pcDNA3.1, row 1), MYC-Rev alone (row 2), Nullbasic (NB)-mCherry alone (row 3 a) or MYC-Rev with NB-mCherry (row 4). Fixed cells were immunostained with anti-MYC (green) and anti-DDX1 (magenta, upper panels) and were visualized alone with NB-mCherry (red) by fluorescence microscopy. Nuclei were stained with DAPI. The overlay panels show the merge of the Rev panel with DDX1 (row 1, 2, and 4) and the NB-mCherry panel with DDX1 panel (row 3). Images are representative of at least five fields selected randomly from three independent experiments. **B**. Quantification of nuclear/cytoplasmic fluorescence ratios(Fn/c) for DDX1as described in Methods. Mean of Fn/c ± SD were calculated from 60 HeLa cells from 3 independent experiments.

Our previous studies observed that Nullbasic redistributes not only Rev’s subcellular localization within the cell from the nucleolus to the nucleoplasm and cytoplasm but also certain cellular proteins, including CRM1, B23 and C23, in a Rev-dependent manner [37]. To investigate whether alteration of Rev subcellular localization induced by Nullbasic also affects the subcellular distributions of DDX1, we visualized its subcellular localization in HeLa cells using fluorescence microscopy. In cells transfected with parental pcDNA3.1 plasmid, endogenous DDX1 was primarily found in the nucleus (Figure 4A, row 1), as observed by others [28]. As expected, the localization of MYC-Rev expressed alone was predominantly nucleolar (Figure 4A, row 2). Interestingly, we observed a significant amount of DDX1 relocalized to the cytoplasm when HeLa cells expressed Nullbasic-mCherry either alone (Figure 4A, row 3) or with MYC-Rev (Figure 4A, row 4). To quantify the extent of cytoplasmic redistribution of DDX1 within the cells expressing Nullbasic, we measured the nuclear/cytoplasmic fluorescence ratio (Fn/c). The result showed that Nullbasic significantly (*p* < 0.0005) enriched the level of DDX1 in the cytoplasm compared with in its absence (Figure 4B). Expression of Nullbasic-mCherry alone or coexpression of Nullbasic-mCherry and MYC-Rev had no apparent effect on DDX3 and DDX17 subcellular distribution [see Additional file 3]. As previous observed [35,37], coexpression of Nullbasic-mCherry with MYC -Rev induced Rev mislocalization from the nucleolus to the nucleoplasm and cytoplasm (Figure 4A, row 4). These data thus show that Nullbasic associates with DDX1 leading to the alteration of DDX1 subcellular distribution.

### Overexpression of DDX1 rescues Nullbasic-induced Rev subcellular mislocalization

**Figure 5:**
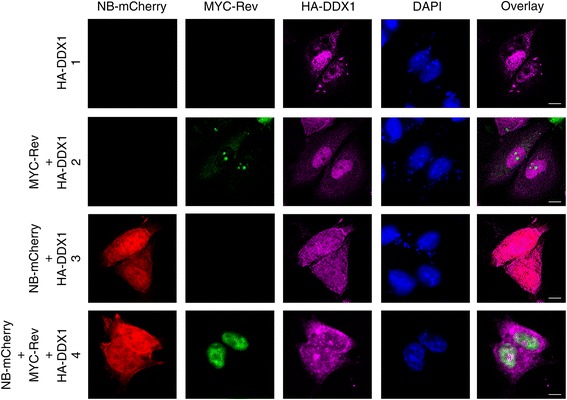
**Overexpression of DDX1 in HeLa cells restores Rev nucleolar localization disrupted by Nullbasic.** HeLa cells were transfected with HA-DDX1 alone (row 1), MYC-Rev with HA-DDX1 (row 2). Nullbasic (NB)-mCherry with HA-DDX1 (row 3) or MYC-Rev with HA-DDX1 and NB-mCherry (row 4). Fixed cells were immunostained with anti-MYC (green) and anti-HA (magenta) antibodies and were visualized alone with NB-mCherry (red) by fluorescence microscopy. Nuclei were stained with DAPI. The overlay panels show the merge of the MYC-Rev panel with the HA-DDX1 panels (row 1, 2, and 4) and the NB-mCherry panel with HA-DDX1 panel (row 3). Images are representative of at least five fields selected randomly from three independent experiments.

Next, we monitored subcellular distributions of MYC-Rev and Nullbasic-mCherry in HeLa cells where DDX1 was overexpressed. We observed that HA-DDX1 was predominantly in the nucleus but some cytoplasmic HA-DDX1 was also detected in the presence or absence of MYC-Rev (Figure 5, row 1 and 2). Expression of Nullbasic-mCherry either alone or with MYC-Rev induced uniform redistribution of HA-DDX1 from the nucleus throughout the entire cell (Figure 5, row 3 and 4). Surprisingly, coexpression of HA-DDX1 with Nullbasic-mCherry resulted in the absence of MYC-Rev in the cytoplasm and its accumulation in the nucleus (Figure 5, row 4), indicating that mislocalization of MYC-Rev to the cytoplasm induced by Nullbasic-mCherry was partly reversed by overexpression of HA-DDX1. Thus, these experiments imply that Nullbasic disrupts a role of DDX1 in maintaining the subcellular distribution of Rev.

### Nullbasic directly binds to DDX1 *in vitro*

**Figure 6:**
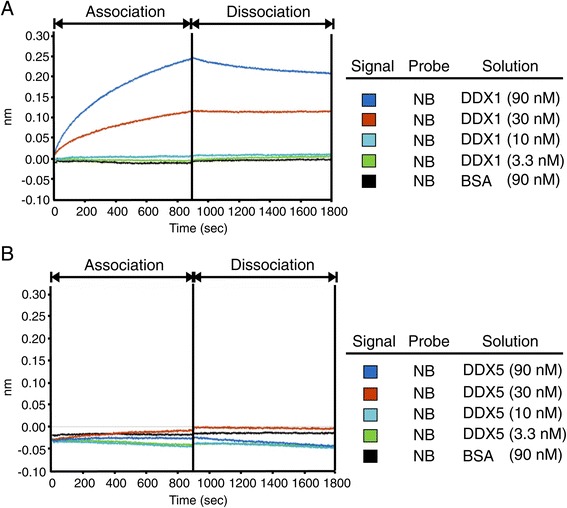
**DDX1 directly interacts with Nullbasic** ***in vitro*** **.** The Octet Red system was used to measure binding events between Nullbasic and DDX1. **A**. BLI sensograms. Biotinylated recombinant Nullbasic-FLAG-V5-6 × His was bound to a streptavidin biosensor and applied to solutions containing 3.3 nM (green), 10 nM (light blue), 30 nM (red) or 90 nM (dark blue) of human recombinant Myc-DDK-tagged DDX1. **B**. The probe was also exposed to DDX5 solutions of the same concentration or with BSA at 90 nM as negative controls. The BLI experiment was repeated 3 times with similar results and a representative sensogram is shown.

Based on the above results, we hypothesized that the RNA helicase DDX1 binds to Nullbasic directly. To test the hypothesis, an *in vitro* binding assay based on Bio-Layer Interferometry (BLI) [40,41] was performed to study the interaction between DDX1 and Nullbasic. Biotinylated recombinant Nullbasic-FLAG-V5-6 × His was immobilized on streptavidin-coated biosensors and incubated with highly purified Myc-DDK-tagged DDX1 obtained from HEK 293 T cells at concentrations ranging from 3.3 to 90 nM. Bovine serum albumin (BSA) was used as a negative control. As predicted, recombinant Nullbasic could directly bind to recombinant DDX1 with a dissociation constant (K_*D*_) value of ~6 nM under the conditions tested, whereas no direct binding was observed between BSA and Nullbasic-FLAG-V5-6 × His (Figure 6A). A second negative control used purified Myc-DDK-tagged DDX5 under the same experimental conditions. The assay showed that DDX5 did not bind to Nullbasic-FLAG-V5-6 × His, even at the highest concentration of 90 nM (Figure 6B). These data indicated that Nullbasic can directly interact with DDX1 *in vitro*.

### DDX1 forms different protein complexes with Nullbasic and Rev in cells

**Figure 7:**
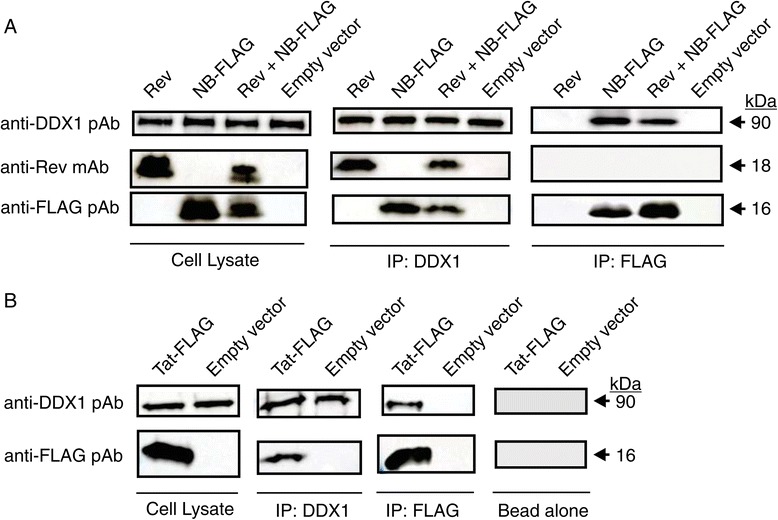
**DDX1 forms different protein complexes with Nullbasic and Rev. A**. HEK293T cells were transfected with either empty vector (pcDNA 3.1) alone, Nullbasic (NB)-FLAG alone, Rev alone or co-transfect NB-FLAG with Rev as indicated. Endogenous DDX1, NB-FLAG and their binding partners were immunoprecipated with anti-DDX1 and anti-FLAG beads. Cell lystes and immunoprecipated proteins were separated by SDS-PAGE and target proteins were detected using anti-FLAG, anti-DDX1 and anti-Rev antibodies. **B**. HEK293T cells were transfected with empty vector or Tat-FLAG as indicated. Target proteins were detected using anti-FLAG, anti-DDX1 after immunoprecipitation using anti-DDX1, anti-FLAG beads or with beads alone. The figures are representative of 3 independent experiments.

Since Nullbasic is able to bind DDX1 directly *in vitro*, co-immunoprecipitation experiments were further undertaken to examine whether Nullbasic can compete with Rev for binding to DDX1. HIV-1 Rev and Nullbasic-FLAG proteins were expressed in HEK293T cells by plasmid transfection either alone or together (Figure 7). After 24 h transfection, cell lysates were used for co-immunoprecipitation experiments. Endogenous DDX1 and Nullbasic proteins were immunoprecipitated with anti-DDX1 and anti-FLAG antibody-coated beads separately. We observed DDX1 co-immunoprecipitated both Rev and Nullbasic, suggesting DDX1 is able to interact with both proteins. However, immunoprecipitation of Nullbasic did not co-immunoprecipitate Rev, as previously reported [37], but did co-immunoprecipitate endogenous DDX1, suggesting that Nullbasic can bind to DDX1 and may form a Nullbasic:DDX1 complex with limited capacity to interact with Rev.

Previous studies have reported that HIV-1 Tat interacted with DDX3, DDX5, DDX18, DDX19, DDX21 and DDX24. However, the interaction between Tat and DDX1 has not been reported. Here, we found that DDX1 associated with HIV-1 Tat in reciprocal immunoprecipitations using either an anti-DDX1 or anti-FLAG as the capture antibody (Figure 7B). These results indicate that the interaction between Nullbasic and DDX1 is not through Tat’s basic domain and Nullbasic can compete with Rev for binding to DDX1.

### Nullbasic inhibition of Rev activity can be counteracted by overexpression of wild type Tat

**Figure 8:**
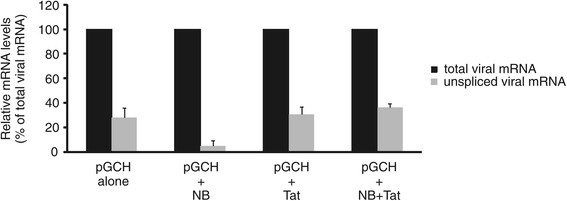
**Overexpression of wild type Tat rescues the unspliced viral mRNA levels suppressed by Nullbasic in HIV-1 expressing cells.** Total RNA was extracted from HEK293T cells transfected to express the HIV-1 infectious provirus, pGCH, and co-transfected at a 1:2 molar ratio with either empty vector (“No Tat”) or plasmids expressing Nullbasic-FLAG (“NB”), Tat-FLAG (“Tat”), or Nullbasic-FLAG with Tat-FLAG (“NB + Tat”). The levels of total (black columns) and unspliced (gray columns) HIV-1 mRNAs were assayed by quantitative RT-PCR using primers specific for total and unspliced viral mRNA. Results represent the means and standard deviations of triplicate assays from 3 independent experiments, with unspliced viral mRNA levels expressed relative to total viral mRNA levels. Significant differences between data were determined using Welch’s *t*-test against a two-tailed distribution, the *p* values from which are indicated.

We previously showed that coexpression of Nullbasic with HIV-1 in producer cells resulted in the reduced steady-state levels of singly-spliced and unspliced viral mRNA [35]. We hypothesize that Nullbasic may act in a dominant-negative manner to disrupt an unrecognized role for Tat in supporting Rev function. To test this hypothesis, we used a recombinant infectious HIV-1 proviral construct, pGCH, in which the cytomegalovirus (CMV) immediate-early promoter replaces the HIV-1 5′ U3 region but retained the natural HIV-1 transcriptional start site in the R region [35]. The pGCH plasmid expresses all accessory and regulatory genes including Tat and Rev. Importantly, RNA polymerase II-directed transcription from the CMV promoter in this construct is only minimally affected by Tat and Nullbasic, thereby allowing the segregation of Tat’s role in transactivation from other potential biological roles in the virus life cycle [35]. As shown in Figure 8, HEK293T cells cotransfected with pGCH and a plasmid encoding Nullbasic-FLAG had significantly reduced (*p* = 0.02) amounts of unspliced viral mRNA relative to total viral mRNA when compared to cells transfected with pGCH alone (Figure 8). Significantly, coexpression of wild type Tat with Nullbasic restored the steady state levels of unspliced viral mRNA (*p* = 0.0005, compared to cells contransfected with pGCH and Nullbasic), suggesting that Tat compensated for the inhibitory effect of Nullbasic on Rev function.

## Discussion

As a general rule, unspliced or incompletely spliced cellular mRNA transcripts, as well as HIV-1 mRNA transcripts that encode many HIV-1 structural and regulatory proteins, are retained in the nucleus and degraded. Extensive research has identified cellular proteins by direct and indirect interactions with Rev that facilitate transport of RRE-containing viral mRNA out of the nucleus [34,42–44]. We had shown that a mutant Tat protein, we call Nullbasic, has an intriguing ability to inhibit Rev function resulting from its mislocalization from the nucleolus to the nucleoplasm and cytoplasm. We hypothesized that Nullbasic targeted and perhaps sequestered a cellular protein, rather than directly targeting Rev as studies have failed to identify interactions between Rev and either wild type Tat [37,45–47] or Nullbasic [37]. To pursue this idea, we used a proteomics-based approach and identified 46 host proteins that co-immunoprecipitated with Nullbasic. Many of the identified proteins can regulate Rev function. For instance, B23 and CRM1 have been previously reported to regulate Rev RNP nucleocytoplasmic transport [4,10] and our previous studies demonstrated that Nullbasic has the capacity to alter the subcellular localizations of B23 and CRM1 in a HIV-1 Rev-dependent manner [37]. In addition to DDX1, DDX3 and DDX17, we also identified DEAH (Asp - Glu - Ala - His)-box helicase 9 (DHX9) as a Nullbasic-interacting protein that has been reported to interact with HIV-1 Rev and mediate Rev RNP complex transport [34,48]. In addition, Matrin 3 and serine/arginine-rich splicing factor 1 (also called ASF/SF2) were previously shown to regulate Rev function. Matrin 3 acts as a cofactor of Rev that binds viral mRNA to form part of the Rev RNP complex and in order to promote the nuclear export and translation of incompletely spliced viral transcript [49–52]. ASF/SF2 is a sequence specific splicing factor involved in pre-mRNA splicing [53]. In order to balance spliced and unspliced forms of HIV-1 transcripts, ASF/SF2 is recruited to the Rev RNP complex and regulates HIV-1 mRNA splicing [54,55]. Our previous study indicated that Nullbasic could reduce steady state levels of incompletely spliced viral mRNA [35], suggesting that Nullbasic may play an inhibitory role in Rev-mediated mRNA splicing or mRNA transport. In this study, we use a Rev-dependent reporter, pGag-RRE, to investigate the roles of identified DEAD-box RNA helicases in Rev function (Figure 3). Because this reporter only contains a 5′splice donor site without a splice acceptor site, transcripts produced from the reporter will accumulate in the nucleus and are efficiently exported to the cytoplasm for gene expression only in the presence of Rev [27]. Our Rev reporter results demonstrated that Nullbasic significantly reduced Rev-dependent reporter gene expression (Figure 3), suggesting that Nullbasic inhibits Rev-mediated mRNA transport function leading to reduced levels of unspliced transcripts, resulting in loss of gene expression. However, we cannot exclude the possibility that Nullbasic also affects HIV-1 mRNA splicing by, for example, interfering with ASF/SF2 function.

The expression level of DDX1 in cells has been shown to highly regulate HIV-1 Rev’s subcellular distribution. In the HeLa, Cos-1 and 293 T cell lines, HIV-1 Rev predominantly localizes in the nucleolus but also to the nucleoplasm to a lesser extent [26,28,33,35,37]. Whereas when DDX1 is expressed at lower levels, for instance in human astrocytes and DDX1-downregulated cells, Rev subcellular distribution is altered the nucleus to cytoplasm [26,28]. Thus DDX1 has been suggested to be required for proper subcellular localization of HIV-1 Rev. By combining our results and above findings, we propose a potential mechanism for the effects of Nullbasic on HIV-1 Rev transport. In order to traffic Rev between the nucleus and cytoplasm, Rev forms protein complexes with different cellular proteins. In the absence of the RRE, Rev, DDX1, B23 and other cellular proteins form a nuclear complex. Our study provides further evidence that a major role for DDX1 is to maintain the stability of this nuclear complex and to thereby ensure Rev’s proper localization. Within cells where Nullbasic is overexpressed, a DDX1 interaction with Nullbasic can modestly shift DDX1 subcellular localization (Figure 4). Our results can be described by a model where Nullbasic competes with Rev for association with DDX1 although alternative explains are possible. For example, the Nullbasic:DDX1 complex may sequester an as yet unknown cellular factor required for Rev nuclear localization. Our study found Nullbasic interaction with DDX1 was Rev-independent and sufficient to dysregulate the subcellular localization of Rev leading to its accumulation in the cytoplasm so that Rev is observed in the cytoplasm and nucleus. When Nullbasic and exogenous DDX1were co-expressed in cells, excessive DDX1 overcame Nullbasic’s inhibitory effect on Rev trafficking thereby stabilizing Rev nuclear localization and function, despite nucleolar localization not being completely restored.

Comparison of Tat and Rev cellular interacting partners using the HIV-1, Human Protein Interaction Database (http://www.ncbi.nlm.nih.gov/RefSeq/HIVInteractions) revealed that many host cell proteins interact with both viral proteins. Moreover, Gautier et al. [56] and Naji et al. [34] used recombinant GST-Tat and MBP-Rev fusion proteins to investigate the *in vitro* host cell interactome of Tat and of Rev, respectively. Gautier et al. identified 183 potential Tat binding partners, of which DDX3, DDX5, RHA, B23, matrin 3, CRM1, eEF1, HNRNPF, HNRNPM, importin α, importin β, adenosine deaminase (ADAR) and poly(A) binding protein (PABPC1) were also detected in Naji’s Rev study. Most of the proteins commonly identified in both studies are essential for the formation of the HIV-1 Rev RNP complex and were also identified in our study. The function of HIV-1 Tat in the nucleolus remains uncertain. Using quantitative proteomics, a study from Jarboui et al. [57] reported that the composition of the nucleolus is changed in cells expressing HIV-1 Tat, indicating that Tat may contribute to creating a cellular environment in the nucleolus suitable for HIV-1 replication. Moreover, we also found that Tat could associate with DDX1 in cells (Figure 7B), and overexpression of wild type Tat could restore Rev activity in cells co-expressing Nullbasic and HIV-1 (Figure 8). One possibility is that Nullbasic is actually acting as a type of transdominant negative protein, implying that wild type Tat plays an undiscovered role in supporting Rev activity in the nucleus.

## Conclusions

Taken together, our work supports the findings that DDX1 is important for proper HIV-1 Rev nuclear localization and function. Nullbasic, a HIV-1 Tat transdominant mutant, sequesters DDX1 in cells resulting in disruption of Rev subcellular distribution and function.

## Methods

### Plasmid constructs

pCMV∆R8.91 was a gift from Andreas Suhribier, QIMR Berghofer Medical Research Institute, Australia and pCMV-VSV-G was from Ian Mackay, The University of Queensland, Australia. A plasmid expressing the BRU clone of HIV-1 Rev (pRSV-Rev) was a gift from Damian Purcell, The University of Melbourne, Australia. pcDNA3-HA, pcDNA3-HA-DDX1, pcDNA3-HA-DDX3 and pcDNA3-HA-DDX17 were obtained from Yasuo Ariumi, Kumamoto University, Japan [33]. pGag-RRE and pGag-CTE were obtained from Hans-Georg Krausslich. The pcDNA3.1-Tat-FLAG plasmid was a gift from Monsef Benkirane, Institute de Génétique Humaine, France. The luciferase expression plasmid, pRL-SV40, was obtained from Addgene (plasmid #27163). The construction of the pcDNA 3.1-Nullbasic-FLAG, pcDNA 3.1-Nullbasic-FLAG-mCherry (termed Nullbasic-mCherry) pcDNA 3.1-Rev and pGCH plasmids have been described previously [35,37]. pLOX-CW-FLAG-mCherry and pLOX-CW-Nullbasic-mCherry expression plasmids were generated by inverse PCR of FLAG-mCherry and Nullbasic-mCherry form pcDNA 3.1-Nullbasic-mCherry, which were then inserted into the pLOX-CW-eGFP plasmid (obtained from Addgene, plasmid #12241) via Bam HI and Sal I restriction sites. The primers used to generate FLAG-mCherry were as follows: Forward (Fwd) 5′-AAA AGG ATC CCC ACC ATG GAC TAC AAG GAC GA-3′ and Reverse (Rev) 5′-TAT GAG TCG ACG TCG CGG CCG CTT TAC-3′. The primers used to generate Nullbasic-mCherry were as follows: Fwd 5′-ACT TAG GAT CCC CAC CAT GGA GC-3′ and Rev 5′-TAT GAG TCG ACG TCG CGG CCG CTT TAC-3′.

### Cell lines and lentivirus particle production

HEK293T and HeLa cells were cultured in Dulbecco’s Modified Eagle medium (DMEM) (Life Technologies) supplemented with 10% (v/v) fetal bovine serum (Life Technologies) and 1% (v/v) penicillin-streptomycin. All cells were typically incubated at 37°C in a humidified 5% CO_2_ atmosphere.

5 × 10^6^ HEK293T cells were cultured in 10-cm dish and co-transfected with 6 μg of pCMV∆R8.91 plasmid, 2 μg of pCMV-VSV-G and 2 μg of pLOX/CW-Nullbasic-mCherry or pLOX/CW-FLAG-mCherry using the standard calcium phosphate transfection method. At 48 h post-transfection, cell culture supernatants containing lentivirus particles were harvested, filtered through 0.45 μm filters and stored in small aliquots at −80°C until needed. The amount of virus in the supernatants was determined using the RETROtek HIV-1 p24 antigen enzyme-linked immunosorbent assay (ELISA) kit (Zeptometrix Corporation) according to the manufacturers’ instructions.

### Co-immunoprecipitation assays

2.5 × 10^6^ HEK293T cells grown in 10 cm^2^ dishes for 24 h and were transfected with of Tat-FLAG (2 μg), Nullbasic-FLAG (2 μg), Rev (2 μg) or cotransfected with Nullbasic-FLAG (2 μg) and Rev (2 μg) using X-tremeGENE HP DNA transfection reagent (Roche) according to the manufacturers’ instructions. After 24 h transfection, the cells were washed once with PBS, trypsinized and harvested by centrifugation for 5 min at 200 × g. The supernatant was discarded and the pellet was lysed at 4°C for 30 min with lysis buffer (50 mM Tris–HCl, pH 7.4; 150 mM NaCl; 1 mM EDTA; 0.5% [v/v] Triton X-100; protease inhibitor cocktail [Roche]). The lysates were cleared by centrifugation at 4°C and 12,000 × g for 10 min. FLAG-tagged proteins and interacting proteins were captured using anti-FLAG M2 magnetic beads (Sigma-Aldrich) as recommended by manufacture and eluted with SDS-PAGE sample buffer (125 mM Tris–HCl, pH 6.8; 4% [v/v] sodium dodecylsulfate (SDS); 20% [v/v] glycerol; 0.004% [w/v] bromphenol blue). Protein lysates were also immunoprecipitated with mouse DDX1 primary antibody (Santa Cruz) conjugated to protein G magnetic beads (Sigma-Aldrich) as recommended by manufacture and eluted with SDS-PAGE sample buffer. The eluted protein complexes were analysed by Western blot using antibodies as described.

### Western blot analyses

Cell lysate were boiled in SDS-PAGE sample buffer and separated by 12% sodium dodecylsulfate – polyacrylamide gel electrophoresis (SDS-PAGE). Gels were electro-blotted onto a polyvinylidene fluoride (PVDF) membrane (Pall) using a semi-dry transfer system (Bio-Rad Laboratories). Nullbasic-FLAG and Tat-FLAG proteins were detected with a rabbit anti-DYKDDDDK Tag polyclonal antibody (Cell Signaling Technology). Rev was detected with a mouse anti-Rev monoclonal antibody (Santa Cruz Biotechnology). Endogenous CDK9, β-actin, PARP, DDX1, DDX3 and DDX17 were detected with a rabbit anti-CDK9 monoclonal antibody (Cell Signaling Technology), mouse anti-β-actin monoclonal antibody (Sigma Aldrich), mouse anti-PARP monoclonal antibody (Biolegend), rabbit anti-DDX1 polyclonal antibody (Abcam), rabbit anti-DDX3 polyclonal antibody (Cell Signaling Technology)) and mouse anti-DDX17 monoclonal antibody (Santa Cruz Biotechnology). Primary antibodies were detected with HRP-conjugated goat anti-rabbit or horse anti-mouse antibodies (Cell Signaling Technology).

### Immunofluorescence analyses and quantification of Fn/c ratios

HeLa cells were grown on glass coverslips, seeded in 6-well plates (2 × 10^5^ cells/well) and transfected were transfected with Rev (1 μg) alone, NB-mcherry alone (1 μg) or cotransfected Rev (1 μg) with NB-mcherry (1 μg) using X-tremeGENE HP DNA transfection reagent according to the manufacturers’ instructions. For DDX1 overexpression experiments, HeLa cells were grown on glass coverslips and transfected with HA-DDX1 (2 μg) alone, Rev (1 μg) with HA-DDX1 (2 μg), NB-mCherry (1 μg) with HA-DDX1 (2 μg) or Rev (1 μg) with NB-mCherry (1 μg) and HA-DDX1 (2 μg) using X-tremeGENE HP DNA transfection reagent.

After 24 h transfection, cells were fixed in 3% (w/v) paraformaldehyde at room temperature for 10 min and quenched with 50 mM NH_4_Cl for 5 min. Cells were then permeabilized with 0.1% (v/v) Triton X-100 for 15 min and blocked in 10% (v/v) normal goat serum (Sapphire Bioscience) for 15 min. Endogenous DDX1 protein was detected with a rabbit anti-DDX1 polyclonal antibody. HA-DDX1 protein was detected with rabbit anti-HA monoclonal antibody. MYC-Rev was probed with mouse anti-MYC monoclonal antibody (Cell Signaling Technology). Primary antibodies were detected with FITC-conjugated goat anti-rabbit antibodies (Life Technologies) and Cy5-conjugated goat anti-mouse antibodies (Life Technologies). Nuclei were stained with 1 μM 4′,6-diamidino-2-phenylindole (DAPI, Life Technologies). Finally, coverslips were mounted onto slides with ProLong Gold antifade reagent (Life Technologies). Fluorescent images were captured using a Leica TCS SP2 confocal scanning microscope (Leica Microsystems) with 63 × objective lenses and standard lasers and filters for FITC, mCherry, Cy5 and DAPI fluorescence. Confocal images were analyzed using the imageJ software 1.48 (http://imagej.nih.gov/ij/) to quantify the fluorescence of nuclear (Fn) and cytoplasmic (Fc). The nuclear/cytoplasm fluorescence ratio (Fn/c) was calculated using the formula, Fn/c = (Fn – Fb) / (Fc – Fb), where Fb is the background fluorescence.

### Rev reporter assays

6 × 10^5^ HEK293T cells were transfected with appropriate expression vectors using X-tremeGENE HP DNA transfection reagent according to the manufacturers’ instructions. To monitor plasmid transfection efficiency, a luciferase reporter plasmid was included in each transfection. After 24 h transfection, the cells were washed with PBS, trypsinized and harvested by centrifugation for 5 min at 200 × g. The pelleted cells were then lysed at 4°C for 30 min with lysis buffer (50 mM Tris–HCl, pH 7.4; 150 mM NaCl; 1 mM EDTA; 1% [v/v] Triton X-100; protease inhibitor cocktail [Roche]). Cell lysates were centrifuged at 12,000 × g for 10 min and clarified supernatants were collected. The amount of p24 was measured by RETROtek HIV-1 p24 antigen ELISA kit according to the manufacturers’ instructions and the transfection efficiencies were assayed by BioLux luciferase assay kit (New England BioLabs) according to the manufacturers’ instructions.

### RNA splicing assay

HEK293T cells were transfected with 0.1 μg of pGCH provirus along with 0.2 μg of either Tat-FLAG or Nullbasic-FLAG plasmids or both. Total RNA was extracted 24 h post-transfection using TRIzol reagent (Invitrogen) and further isolated using the PolyAtract mRNA isolation system (Promega). The purified total RNA was reverse transcribed to cDNA using random hexamer primers and M-MuLV RT (New England BioLabs) according to the manufacturer’s instructions. Viral cDNAs were quantified by quantitative PCR using Platinum SYBR Green Supermix (Invitrogen) on the Rotor-Gene Q (Qiagen). The primers used to detect total and unspliced viral mRNA has been described previously [58]. Reverse-transcribed GAPDH cDNA was used to normalize extraction efficiency.

### Recombinant proteins

Recombinant DDX1 and DDX3 proteins were purchased from OriGene. The recombinant Nullbasic-FLAG-V5-6xHis was produced in *E. coli* strain BL21-AI (Invitrogen) transformed by pDEST42-Nullbasic-FLAG. A 200 ml culture was grown in Luria broth to log phase and induced with 0.2% arabinose and 1 mM IPTG. After 4 hours the E. coli was collected by centrifugation and immediately processed. 1× FastBreak™ Cell Lysis Reagent (Promega) was added to the cell pellet for 15 min and the lysate was mixed with 2 ml of Chelating Sepharose beads (GE Healthsciences) for 1 min. The beads were placed into a column and wash buffer (100 mM HEPES pH7.5, 10 mM imidazole) was applied 6 times. Two ml of elution buffer (100 mM HEPES pH7.5, 500 mM imidazole) was added and after 1 min was collected. The eluted protein was dialysed twice for 90 minutes each in 1 liter of storage buffer (20% glycerol v/v, 50 mM Tris–HCl pH8.0, 1 mM ZnCl_2_, adding fresh 2 mM 1,4-Dithioerythritol every 30 minutes). The protein was stored in aliquots under liquid nitrogen.

### Bio-Layer interferometry assay

Interaction assays were performed in black 96 well microplates (Greiner Nio-one) at 25°C using Octet RED96 instrument (ForteBio). The recombinant Nullbasic proteins were biotinylated using EZ-Link Sulfo-NHS-Biotin (Pierce Biotechnology) following the manufacturer’s instruction. Biotinylated Nullbasic proteins (1 μM) were loaded onto Streptavidin biosensors (ForteBio) for 15 min in 1× kinetics buffer (1 mM phosphate, 150 mM Nacl, 0.002% Tween-20 and 0.1 mg/ml BSA). Load biosensors were then washed in 1× kinetics buffer at a shake speed of 1000 rpm for 5 min. Association was monitored by transferring ligand biosensors to wells containing recombinant DDX1 or DDX3 with concentrations of 3.3 to 90 nM in 1× kinetics buffer at a shake speed of 1000 rpm for 15 min followed by disassociation in 1× kinetics buffer at a shake speed of 1000 rpm for 15 min. Data were processed using Octet data analysis software 7.0 (ForteBio). Interaction analysis between Nullbasic and BSA was also included as a negative control.

### Generation of HeLa-based cell line stably expressing FLAG-mCherry and Nullbasic-mCherry

HeLa cells were transduced with 200 ng CAp24 of lentivirus prepared as described above. After 24 h transduction, cells were washed, replaced with new culture medium and further incubated for 48 h. At 72 h post-transduction, cells were trypsinized, filtered through 37 μm Nylon Mesh to remove cellular clumps and diluted in PBS to a concentration of 2 × 10^7^ cells/ml. Fluorescence-activated cell sorting (FACS) analyses were performed to isolate cells expressing high levels of mCherry using a MoFlo high speed cell sorter (Beckman Coulter). The isolated HeLa cells expressing Nullbasic-mCherry (Hela-Nullbasic-mCherry) or FLAG-mCherry (Hela-FLAG-mCherry) were expended and prepared for proteomic experiments.

### Preparation of nuclear extracts

Preparation of nuclear extracts from Hela cells was performed as described previously [59,60]. Briefly, 1.8 × 10^7^ Hela-Nullbasic-mCherry and Hela-FLAG-mCherry cells were trypsinized and harvested by centrifugation for 10 min at 1850 × g. The pelleted cells were resuspended in five volumes of 4°C PBS and collected by centrifugation for 10 min at 1850 × g. The pelleted cells were resuspended in five packed cell volumes (pcv) of hypotonic buffer (10 mM HEPES, pH 7.9; 1.5 mM MgCl_2_; 10 mM KCl; 0.5 M DTT; protease inhibitor cocktail) and collected by centrifugation. The packed cells were resuspended in three pcv of hypotonic buffer and allowed to swell for 10 min. The cells were transferred to a glass Dounce homogenizer tube and lysed by 10 up-and-down strokes using a clearance pestle. The homogenate was transferred to a new centrifuge tube and centrifuged for 15 min at 3300 × g. The pellet obtained from the centrifugation of the homogenate was subjected to a second centrifugation for 20 min at 25,000 × g to remove residual cytoplasmic material and this pellet was designated as crude nuclei. The crude nuclei were resuspended in one packed nuclear volume (pnv) of low salt buffer (20 mM HEPES, pH 7.9; 25% (v/v) glycerol; 1.5 mM MgCl_2_; 0.2 mM EDTA; 0.5 M DTT; protease inhibitor cocktail) and transferred to a glass Dounce homogenizer tube. The nuclei were stroked gently using a type B pestle. While stocking, 5 M KCl was added to the resuspended nuclei drop wise to make the final concentration 0.42 M. The extract was transferred to a capped tube and mixed gently on a rotating platform for 30 min at 4°C. The nuclear extract collected by ultracentrifugation for 1 h at 100,000 × g using Beckman Coulter SW41T1 rotor and stored at −80°C. The total protein concentrations of nuclear fractions were determined by the Bradford method [61] against a bovine serum albumin standard.

### In-gel tryptic digestion

FLAG-tagged proteins and interacting proteins from nuclear extracts were precipitated 2 h at 4°C with 40 μl of Anti-FLAG Beads (Clontech). Immunoprecipitates were spun down at 5000 × g for 1 min, washed three times with wash buffer (50 mM Tris–HCl, pH 7.4; 150 mM NaCl; 1 mM EDTA; protease inhibitor cocktail) and eluted by competition with 200 μg/ml of FLAG peptide (Sigma-Aldrich) overnight at 4°C. Following co-immunoprecipitation assays, protein lysates were boiled in SDS-PAGE sample buffer and separated by 12% SDS-PAGE. Gels were stained in Bio-Safe Coomassie stain (Bio-Rad) for 2 h and then washed in H_2_O overnight. Protein bands were observed and images were generated by a LAS500 imaging system (Fujifilm Life Science). Protein bands of interest were excized and cut into 1 × 1 mm pieces for analysis. Gel pieces were destained in 200 μl of destaining buffer (50% [v/v] CH_3_CN; 200 mM NH_4_HCO_3_) for 90 min and dried with 200 μl of 100% (v/v) CH_3_CN for 10 min. Dried gel pieces were rehydrated with 40 μl of trypsin solution (10% [v/v] CH_3_CN; 40 mM NH_4_HCO_3_; 20 ng/μl Trypsin [Promega]) at 37°C for 1 h. Addition 50 μl of rehydration solution (10% [v/v] CH_3_CN; 40 mM NH_4_HCO_3_) were added and incubated at 37°C overnight. Following incubation, gel pieces were microwaved on a low heat setting for 1 min and peptides were collected into clean 1.5 ml tubes. 100 μl of 5% (v/v) formic acid was added to the gel pieces, which were incubated for 20 min and sonicated three times for 15 sec. Peptides were collected and pooled with those collected previously. Next, 100 μl of formic acid/acetonitrile solution (1% [v/v] formic acid; 60% [v/v] CH_3_CN) was added, and the gel pieces incubated for 5 min and sonicated 3 times for 15 sec. Peptides were collected and pooled with those collected previously. The pooled peptides were dried in the miVac concentrator (Genevac) and stored at −80°C until required. Before analysis by mass spectrometry, dried peptides were resuspended in 25 μl of 1% formic acid.

### Protein identification by mass spectrometry

Following in-gel tryptic digestion, samples were analysed by LC-MS/MS on a Nano HPLC (Shimadzu) coupled to a QStar Elite mass spectrometer (AB SCIEX) with a nano-electrospray ion source. Before sample injection, 50 mm × 300 mm C_18_ trap columns (Agilent) were equilibrated with 1% Solvent B (90% [v/v] CH_3_CN; 0.1% [v/v] formic acid). 8 μl of each extract was injected into an equilibrated C_18_ trap column at 30 μl/min flow rate. Samples were desalted on the column for 6 min and then aligned with the nano HPLC 150 mm x 300 mm C_18_ column (Vydac) for mass spectrometry analysis. Peptides were eluted from the column with a linear gradient of 1% to 40% solvent B over 25 min at 3 μl/min flow rate, followed by a steeper gradient from 40% to 80% solvent B over 5 min were used for peptide elution. Mass spectrometry was performed with ion spray voltage set to 3000 V, curtain gas flow 16, nebuliser gas 1 (GS1) and interface heater at 120°C. The mass spectrometer acquired full scan of TOF-MS data followed by a full scan product ion data in an information dependent acquisition mode. Full scan TOF-MS data was acquired over a mass range of 350 – 1800 and 100 – 1800 for product ion ms/ms. Ions observed in the TOF-MS scan exceeding a threshold of 12 counts and a charge state of +2 to +5 were set to trigger the acquisition of product ion, MS/MS spectra of the resultant 3 most intense ions. The data was acquired and processed using Analyst® QS 2.0 software (ABSCIEX).

## Additional files

Additional file 1:
**The purity of nuclear factions.** Fractionated protein extracts were suggested to western blot analysis for determination of the purity of nuclear and cytoplasmic fractions. Antibodies detecting β-actin, poly(ADP-ribose) polymerase (PARP) and FLAG were used. Additional file 2:
**Forty-six proteins consistently detected in nuclear fractions.** Proteins present in Nullbasic-expressed nuclear fraction were identified by mass spectrometry. Proteins were considered “identified” if two or more peptides with > 95% confidence were detected. The identified proteins were grouped by protein function. Additional file 3:
**Nullbasic does not affect the DDX3 and DDX17 subcellular distributions.** HeLa cells were transfected to express empty vector alone (pcDNA3.1, row 1 and 3), MYC-Rev alone (row 2 and 6), Nullbasic (NB)-mCherry alone (row 3 and 7) or MYC-Rev with NB-mCherry (row 4 and 8). Fixed cells were immunostained anti-MYC antibodies (green) with anti-DDX3 or DDX17 antibodies (magenta) before the subcellular localization of MYC-Rev, NB-mCherry (red), endogenous DDX3 and DDX17 were visualized by fluorescence microscopy. Nuclei were stained with DAPI. Images are representative of at least five fields selected randomly from three independent experiments.
