# Supplementary figures and images for: A HIV-1 Tat mutant protein disrupts HIV-1 Rev function by targeting the DEAD-box RNA helicase DDX1
Source: Retrovirology. 2014 Dec 14;11:121. doi: 10.1186/s12977-014-0121-9 (PMC4271445; doi:10.1186/s12977-014-0121-9)

## Additional File 1

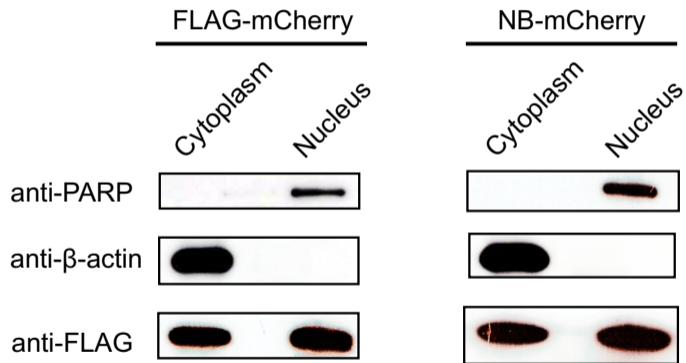

Supplement: Additional file 1: — The purity of nuclear factions. Fractionated protein extracts were suggested to western blot analysis for determination of the purity of nuclear and cytoplasmic fractions. Antibodies detecting β-actin, poly(ADP-ribose) polymerase (PARP) and FLAG were used. [file 12977_2014_121_MOESM1_ESM.pdf]

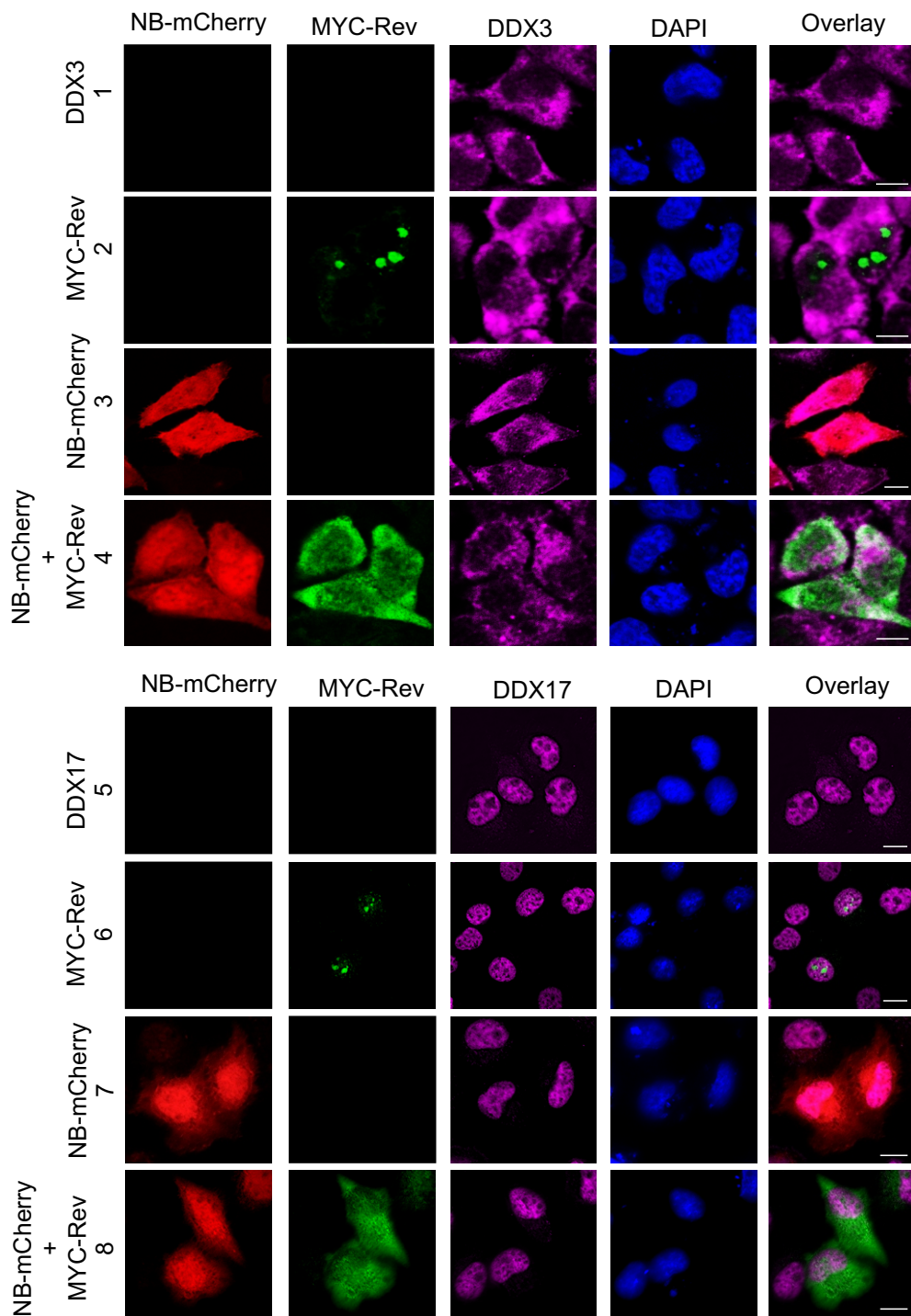

Supplement: Additional file 3: — Nullbasic does not affect the DDX3 and DDX17 subcellular distributions. HeLa cells were transfected to express empty vector alone (pcDNA3.1, row 1 and 3), MYC-Rev alone (row 2 and 6), Nullbasic (NB)-mCherry alone (row 3 and 7) or MYC-Rev with NB-mCherry (row 4 and 8). Fixed cells were immunostained anti-MYC antibodies (green) with anti-DDX3 or DDX17 antibodies (magenta) before the subcellular localization of MYC-Rev, NB-mCherry (red), endogenous DDX3 and DDX17 were visualized by fluorescence microscopy. Nuclei were stained with DAPI. Images are representative of at least five fields selected randomly from three independent experiments. [file 12977_2014_121_MOESM3_ESM.pdf]
