# Supplementary material for: A HIV-1 Tat mutant protein disrupts HIV-1 Rev function by targeting the DEAD-box RNA helicase DDX1
Source: Retrovirology. 2014 Dec 14;11:121. doi: 10.1186/s12977-014-0121-9 (PMC4271445; doi:10.1186/s12977-014-0121-9)
Supplement: Additional file 2: — Forty-six proteins consistently detected in nuclear fractions. Proteins present in Nullbasic-expressed nuclear fraction were identified by mass spectrometry. Proteins were considered “identified” if two or more peptides with > 95% confidence were detected. The identified proteins were grouped by protein function. [file 12977_2014_121_MOESM2_ESM.pdf]

Host protein	Gene symbol	No. times identified[a]	Molecular weight (Da)	No. peptides[b]
RNA processing (binding, splicing and transport)				
ATP-dependent RNA helicase DDX3	DDX3	3	73271	20
Probable ATP-dependent RNA helicase DDX17	DDX17	3	80457	31
ATP-dependent RNA helicase A (RHA)	DHX9	3	140958	21
ATP-dependent RNA helicase DDX1	DDX1	3	82432	11
Ras GTPase-activating protein-binding protein 1	G3BP1	2	52164	2
RNA-binding protein 14	RBM14	3	69492	18
RNA-binding protein FUS	FUS	3	53426	6
Heterogeneous nuclear ribonucleoprotein M	HNRNPM	3	77516	22
Matrin 3	MATR3	3	94623	24
Fragile X mental retardation syndrome-related protein 2	FXR2	3	74223	2
U1 small nuclear ribonucleoprotein A	SNRPA	2	31279	23
U2 small nuclear ribonucleoprotein A	SNRPA1	2	28416	23
Serine/arginine-rich splicing factor 1 (ASF/SF2)	SRSF1	2	27745	23
Serine/arginine-rich splicing factor 2	SRSF2	2	25476	10
Serine/arginine-rich splicing factor 8	SRSF8	2	32288	5
Heterogeneous nuclear ribonucleoprotein H3	HNRNPH3	2	36926	9
Polyadenylate-binding protein 1	PABPC1	2	70671	12
Poly(rC)-binding protein 1	PCBP1	2	37498	8
Poly(rC)-binding protein 2	PCBP2	2	38580	6
Fragile X mental retardation syndrome-related protein 1	FXR1	2	69721	1
Polypyrimidine tract-binding protein 1	PTBP1	2	57221	6
SAP domain-containing ribonucleoprotein	SARNP	2	23671	7
Protein transport/folding				
Nucleophosmin (B23)	NPM1	3	25049	17
Exportin-1 (CRM1)	XPO1	3	123386	3
Transferrin receptor protein 1	TFRC	2	84871	1
Heat shock 70 kDa protein 8 (HSC70)	HSPA8	3	70898	25
Heat shock 70 kDa protein 1A (HSP72)	HSPA1A	2	70052	23
Transcription				
Cyclin T1	CCNT1	3	80685	4
Cyclin dependent kinase 9	CDK9	3	42778	11
TATA-binding protein-associated factor 15	TAF15	2	61830	2
X-ray repair cross-complementing protein 6	XRCC6	2	69843	6
Zinc finger protein 326	ZNF326	2	65654	6
TAR DNA-binding protein 43 (TDP43)	TARDBP	2	44740	14
Translation				
Eukaryotic translation elongation factor 1-alpha	eEF1A	2	50470	8
Eukaryotic translation elongation factor 1alpha-like 3	eEF1A1P5	2	50185	13
Cell organisation and cytoskeleton				
Myosin-9	MYH9	3	226532	5

LIM domain and actin-binding protein 1	LIMA1	3	85226	9
Filamin-A	FLNA	3	280739	13
Filamin-B	FLNB	2	278195	13
Guanine nucleotide-binding protein subunit beta-2-like 1	GNB2L1	2	35077	19
F-actin-capping protein subunit beta	CAPZB	2	31350	15
Drebrin	DBN1	2	71429	5
Spectrin beta chain, brain 1	SPTBN1	2	274609	1
Catalytic activity				
Serine/threonine-protein phosphatase PP1-alpha catalytic subunit	PPP1CA	2	37512	34
Serine/threonine-protein phosphatase PP1-beta catalytic subunit	PPP1CB	2	37187	27
Glyceraldehyde-3-phosphate dehydrogenase	GAPDH	2	36054	24

a: number of times of proteins was identified (out of 3 independent experiments).

b: average number of peptides from the protein sequence detected by mass spectrometry with >95 % confidence.
